# Phenylalanine Is Required to Promote Specific Developmental Responses and Prevents Cellular Damage in Response to Ultraviolet Light in Soybean (*Glycine max*) during the Seed-to-Seedling Transition

**DOI:** 10.1371/journal.pone.0112301

**Published:** 2014-12-30

**Authors:** Joe H. Sullivan, DurreShahwar Muhammad, Katherine M. Warpeha

**Affiliations:** 1 Department of Plant Science and Landscape Architecture University of Maryland, College Park, Maryland, United States of America; 2 Molecular, Cell and Developmental Program, Department of Biological Sciences, University of Illinois at Chicago, Chicago, Illinois, United States of America; University of Texas at Austin, United States of America

## Abstract

UV-radiation elicits a suite of developmental (photomorphogenic) and protective responses in plants, but responses early post-germination have received little attention, particularly in intensively bred plants of economic importance. We examined germination, hypocotyl elongation, leaf pubescence and subcellular responses of germinating and/or etiolated soybean (*Glycine max* (L.) Merr.) seedlings in response to treatment with discrete wavelengths of UV-A or UV-B radiation. We demonstrate differential responses of germinating/young soybean seedlings to a range of UV wavelengths that indicate unique signal transduction mechanisms regulate UV-initiated responses. We have investigated how phenylalanine, a key substrate in the phenylpropanoid pathway, may be involved in these responses. Pubescence may be a key location for phenylalanine-derived protective compounds, as UV-B irradiation increased pubescence and accumulation of UV-absorbing compounds within primary leaf pubescence, visualized by microscopy and absorbance spectra. Mass spectrometry analysis of pubescence indicated that sinapic esters accumulate in the UV-irradiated hairs compared to unirradiated primary leaf tissue. Deleterious effects of some UV-B wavelengths on germination and seedling responses were reduced or entirely prevented by inclusion of phenylalanine in the growth media. Key effects of phenylalanine were not duplicated by tyrosine or tryptophan or sucrose, nor is the specificity of response due to the absorbance of phenylalanine itself. These results suggest that in the seed-to-seedling transition, phenylalanine may be a limiting factor in the development of initial mechanisms of UV protection in the developing leaf.

## Introduction

Seedling establishment is a critical period in the life cycle of any plant, where many abiotic signals are experienced and the seedling must quickly acclimate, yet few studies have investigated the range of seedling responses to UV at early developmental stages [1], [2]. The signal response cascade following absorption by a single or multiple photoreceptor(s), still poorly understood across the many UV wavelengths [3]–[7], and is not well studied outside of *Arabidopsis thaliana*.

Synthesis and deployment of UV screening pigments is presumably an adaptive response, increases epidermal screening of UV-B radiation, and has been demonstrated for the last four decades [1], [3], [8]. Searles *et al.*, (2001) found in a meta-analysis that the accumulation of phenolic-based UV-screening compounds was the most common response of plants to UV-B radiation, so phenylalanine (Phe) may be a key determinant of any such response [9].

Phe-derived phenylpropanoids such as flavonoids [10] and simple phenolics such as the hydroxycinnamic acids and cinnamate esters can absorb UV wavelengths [11]–[13]. Flavonoids and other UV-screening pigments are found in seeds, which vary with regard to both composition and concentration among different plant species [14]. The rate-limiting step in general for phenylpropanoids is the synthesis or availability of Phe [15]–[17]. In seedlings of *Arabidopsis thaliana*, it was demonstrated that Phe supply was important to survival against UV-C, and in pigment synthesis after exposure to UV-A and UV-B wavelengths [3]. While UV-C does not reach sea-level, this assay was key in demonstrating the importance of Phe and phenylpropanoids in high-energy UV-protection.

Variations in environmental conditions can shape or modulate plant biochemistry, physiology, anatomy, development and productivity. Plants have evolved protective and/or repair mechanisms that both detect and provide tolerance to extremes or ranges of environmental conditions. Inability to respond to abiotic signals may have a negative impact on one or more aspects of plant development or physiology, and potentially affect the plant life cycle by reducing biomass or seed yield. Ultraviolet-B (UV-B) radiation between 280 and 320 nm has been studied as a plant ‘stress’ for several decades with the highest level of interest arguably occurring in the 1980’s and 1990’s when depletion of the stratospheric ozone layer and its impacts on plants was poorly understood. Advances in scientific knowledge have increased our understanding of the atmospheric processes involved in ozone formation and depletion, and the current projection is that ozone levels will return to 1980 levels by mid-century [18]. Penetration of UV-B is greater in herbaceous dicotyledonous plants [19], which includes many plants of economic importance. Hence it is important to reveal the strategic locations within leaf tissue that structures may be developed or synthesized in the still poorly understood reception of UV radiations.

Understanding the biochemical and physiological mechanisms of UV acclimation and tolerance remain of interest from a mechanistic point of view and for plants of economic importance. Exposure to high levels of UV-B can have negative effects on photosynthetic capacity, biomass and seed yield, nutritional quality of the seed, altered patterns of species competition, plant ultrastructure, pigment production, reduced genomic stability and increased susceptibility to disease [20], [21]. However many plants experience developmental responses to UV-B as well (reviewed in [22]) and can be very tolerant to even high levels of UV-B. Plants exhibit inter- and intra-specific differences in UV-B tolerance [21], [23]–[26], where tolerance can vary with phenological stage of development and under contrasting environmental conditions [1], [27]. For example, responses vary when the spectral balance of UV-B to the other portions of the solar spectrum is altered [21], [28], [29]. Variations in other environmental factors such as water or nutrient availability may also alter the plant’s response to UV radiation. Therefore, it is difficult to partition responses across multiple environmental factors. Here, we examine how Phe may attenuate seedling responses to UV-A and UV-B in the absence of other confounding environmental factors in germinating soybean (*Glycine max* (L.) Merr.).

Soybean is an important intensively bred agricultural crop that has been often studied for its responses to UV-B [23], [26], [28], [30]–[36]. However, little is known of the spectral sensitivity of the responses during emergence/early seedling establishment. At germination and in the first days after a seedling may experience UV, but may not have a fully functioning chloroplast, and is still dependent upon carbon and phenylpropanoid contents of the seed. Responses of young seedlings to varying levels of UV have been shown to affect overall sensitivity and growth [1], [2], [37]. Moreover, the ‘sprouts’ industry for soybean and other crops of economic importance is growing, and the interest in phenylpropanoids for their application to human health is growing [38], [39]. Therefore, understanding the balance of damage and defense mechanisms, and spectral sensitivity of the UV response in young seedlings under controlled conditions will permit better understanding of plant perception and response to UV in the natural environment.

We have investigated responses that begin in the seed and the responses of the first primary leaves in young soybean to different wavelengths of UV spanning UV-B and UV-A spectral regions, with the hypothesis that UV-B may incur damage, and UV-A development. We were surprised that the UV-B and UV-A effects were not so clear-cut, with UV-B inducing developmental responses in some physiological contexts and damage in others. Phe is known to be important responder to UV, but it appears to serve in multiple capacities across the UV spectrum, and is dependent upon the tissue and developmental context. We propose a strategic role for Phe in post-germination growth and defense, and a specific role of Phe in the development of pubescence optimal for screening UV.

## Materials and Methods

### Plant materials and accessions

*Glycine max* L. seeds of the Williams 82 (Maturity Group IV) cultivated variety were originally obtained from Dr. William Kenworthy (University of Maryland). Seeds of Harosoy (maturity Group II) corresponding to isogenic lines L62-561 (glabrous) and L62-801 (dense) were obtained from Dr. Randall Nelson (National Soybean Research Center, University of Illinois at Urbana).

### Chemicals

All chemicals unless specifically described otherwise were obtained from Sigma (St. Louis, MO USA).

### Growth conditions and UV treatments

Seeds were surface sterilized for 30 min in 20% bleach, washed well with sterile water three times, then imbibed for 1 h in sterile water in complete darkness. All subsequent steps occurred in a dark room unless otherwise specified, aided only by dim green safelight, to prevent exciting light receptors [40]. Seeds were then sown on 0.8% agarose plates containing 0.5 X Murashige and Skoog media, using low-melt “Top” agarose cooled to 50°C, poured to form a very thin (∼1 mm) layer over the seeds (5 mL/phytatray), in order to keep them in place and hydrated [3]. Sucrose was not used in the standard medium (unless otherwise specified), in order not to introduce an additional source of carbon.

Seedlings were grown in complete darkness for either 3 or 7 d before being irradiated with a total fluence of 10^4^ µmol m^−2^. At day (d) 3 after planting, seeds have begun to germinate (i.e. d 1 of growth). So 7 d after planting, seedlings are 4 d-old; 8 d after planting, 5 d-old. Henceforth, the age of the seedling will either be referred to as “germinating seeds” [d 3] or 4 d-old (7 d post sowing), or 5 d-old (8 d post sowing) seedlings.

The UV fluence selected was based on past photobiology work [40] detailing a minimum of incident photons predetermined to give a physiological effect in etiolated seedlings. The photon exposure was selected as a reference point for the purposes of this study and not to simulate sunlight. The total fluence of 10^4^ µmol m^−2^ was controlled by varying exposure times at 300 nm [18 min], 305 nm [16 min], 311 nm [14 min], 317 nm [11 min]), 325 nm [6 min], 332 nm [6 min] or 368 nm [4 min], calculated from lamp output spectra as per [3]; times avoided reciprocity failure.

The UV source was assembled by the UV-B Monitoring and Research Program (UVBMRP), Colorado State University, Fort Collins, CO, USA and consisted of a UV lamp fitted with narrow band pass filters to achieve specific UV-A and UV-B wavelengths, where filters had a 2 nm FWHM at the following center wavelengths: 300, 305, 311, 317, 325, 332 and 368 nm [3]. Control plants were handled exactly as the experimental plants except that a “mock” irradiation was conducted; i.e. no radiation administered during ‘treatment’ [41].

Following irradiation period on day 3, seedlings were either returned to continuous darkness (Dc), or placed in a 16::8 light::dark cycle (W_LD_) at an illumination of 100 µmol m^−2^ s^−1^ of white light (GE Cool-white T12 fluorescent lamps, General Electric, Fairfield, CT USA). Completed germination (breaking testa and emergence of radical) was scored 3 d after irradiation for all seeds, and hypocotyl length (nearest mm) was measured 5 d following irradiation.

### Amino acid-feeding and sucrose experiments

Duplicate sets of seeds were planted similarly to that used in germination studies. For Phe-feeding experiments, Phe was added to low-melt “Top” agarose to a final concentration of 1.0 mM at planting for light-grown experiments. This concentration was chosen partly based on the studies on the *Phe insensitive growth* (*pig1-1*) mutant [42] and was successfully used in [3] as a saturating level of Phe for Arabidopsis. After assessing the data, we repeated the Phe experiments at a lower concentration (500 µM) to avoid exceeding saturation of the response, and an additional series of 1 µM, 10 µM, and 100 µM of Phe were also tested for hypocotyl growth. A number of other amino acids were also tested including the closely related aromatic amino acids tyrosine and tryptophan, added to top agarose at a final concentration of 100 or 500 µM. A separate experiment was done where sucrose (sterile filtered) was added to top agarose (2% sucrose final concentration). The Control (no Phe) included 0.5X MS at the same volume used for amino acids (20-fold dilution). Germinating sets of seedlings (d 3) were irradiated with 10^4^ µmolm^−2^ of UV radiation (300, 305, 311, or 317 nm) or no UV (Control) as previously described, then grown under W_LD_ described above for 5 more d. There were three replicates of each treatment with 30 seeds each, where for each set 10 hypocotyls randomly were selected to be measured to the nearest mm.

### Microscopy

In separate studies, 4-d-old etiolated seedlings grown in complete darkness were treated with UV radiation as described above, returned to darkness for 24 h, then the first developing leaves were harvested and prepared for Scanning Electron Microscopy (SEM), Transmission Electron Microscopy (TEM), deconvoluting, or stereo-dissection microscopy, with SEM, TEM, and deconvoluting methods described previously [3], [41]. The 368 nm wavelength was chosen as it is UV-A, and 317 nm was chosen as it was an expected UV-B wavelength to penetrate the atmosphere, and 300 nm the lower end of UV-B, which does not reach sea level but can penetrate high altitude. For SEM, the cells examined were those of the epidermal layer and the pubescence. Leaf hair length was determined from SEM images by sampling 10 central (side) hairs on each of 10 seedlings. For TEM, the epidermis and first/adjacent mesophyll layer of cells of the primary leaf pair from the abaxial side (first exposed as cotyledon opens) were examined. Fluorescence images were obtained from live (in sterile water) seedling first leaves, 24 h post-UV treatment, on a Zeiss Axiovert 200M microscope (Carl Zeiss; Oberkocken, Germany). The 20X objective was used, and images were obtained with a digital camera utilizing DAPI (blue), FITC (green) and Texas Red (red) filter sets (Chroma; Rockingham, VT) in order to approximately span most of the visible light spectrum. Due to the camera type, images were false colored. At least 30 seedlings were viewed per condition. On the dissecting microscope (Zeiss Stereo Discovery V.8), images of live, immediately removed soybean primary leaves were taken of 5-d-old seedlings (24 h after a treatment of UV, Phe (included from planting), or mock treatment). For Phe applications to 4-d-old seedlings, 1.0 mM Phe in 0.5X MS was poured on (10 mL) and orbitally rotated for 5 min, then permitted to sink in for 1 h, any not absorbed was poured off, then 5 mL 0.5X MS was poured on, rotated, then poured off to remove residue on stem base and any exposed root. Irradiation with 317 nm occurred after the Phe treatment (1.0 h post) for this specific treatment. All seedlings were viewed live, using Axiovision software, and utilizing a DAPI-LongPass filter set. DAPI LongPass excites at a peak of 325 nm (but does excite in both UV-B and UV-A). Images show all visible-spectrum wavelengths as captured by a real color camera. At least 30 seedlings from each treatment were viewed.

### Absorbance spectra

Etiolated seedlings were grown and treated as described for microscopy, using Control, 300, 317, 368 nm treatments, then returned to darkness immediately after treatment. For spectra, 24 h post-irradiation, primary leaf tissue was harvested directly into aqueous buffer (50 mM K_2_PO_4_ pH 7.5, 1.0 mM dithiothreitol, and 0.5% protease inhibitor cocktail for plants (Sigma, St. Louis, MO USA) and ground (30 seedlings) [41]. Cell wall material and organelles were removed by centrifugation. Absorbance of the supernatant was measured using a Perkin-Elmer 7000EL (Perkin Elmer, Waltham, MA, USA) spectrophotometer. Spectra of leaf “hairs” were attained by harvesting whole primary leaves into liquid nitrogen, under dim green lighting. Once frozen, pubescence was dislodged from leaf surfaces with a plastic cell scraper and metal spatula (viewed with dissecting microscope under white light). Leaf material then was lifted out of the liquid nitrogen, with pubescence remaining in mortar to be ground in extraction buffer post-evaporation of liquid nitrogen, processed in the same manner for a spectrum, described above.

### Phenylpropanoid quantitation

Briefly, four sets of seeds (30) were planted in complete darkness. 4-d-old seedlings (d 7) were exposed to 317 nm or a mock (control) irradiation as described for earlier experiments. Aerial portions were harvested directly into liquid nitrogen under dim green light 24 h later. Once frozen, hairs were scraped into liquid nitrogen, and leaf material removed, as described above for absorbance spectra. Sample preparation for analysis was similar to a published procedure [41], except final dried samples were further purified by dissolving in water, then extracted with ethyl acetate and analyzed by LC-MS [43]. Multiple-reaction motoring method in negative ion mode was used for the analysis of particular phenylpropanoids including quercetin, hydroxycinnamate, sinapate, coumarin, with standards obtained from Sigma. Levels determined were expressed as ng/g of liquid-nitrogen-ground-tissue. Liquid-nitrogen-dried/ground-tissue of aerial portions represent 30 pairs of primary leaves each (untreated, UV-treated 317 nm). Identities of structures were confirmed by MS.

### Statistical analysis

Unpaired *t* tests were calculated for groups of seedlings utilizing Prism v.5 software (GraphPad 2011) using Welch’s correction. For appropriate data sets ANOVA also assessed utilizing Prism v.5. 30 seedlings were used per experimental replicate, unless otherwise stated. A minimum of 3 independent replicates were used per experiment. Results were noted as significant if P<0.05.

## Results

### Action spectra of responses indicate differential responses for discrete UV wavelengths; germination and hypocotyl responses indicate unique underlying mechanisms

**Figure 1:**
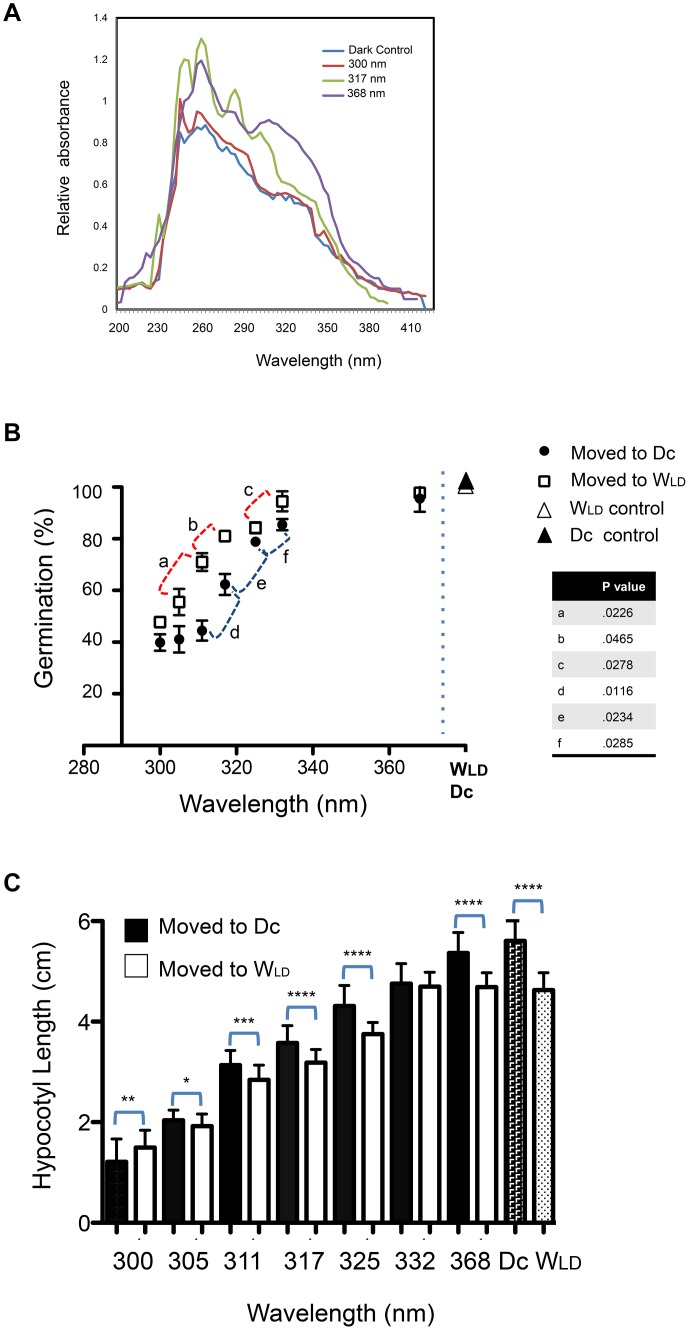
A. Etiolated soybean primary leaf pair spectra in response to UV radiation. Soybean were grown in complete darkness, irradiated with the wavelength (300, 317 or 368 nm) as shown on the Figure, then primary leaf pair extracted for aqueous UV-absorbing molecules. Absorbance was read from 200–420 nm. Spectra are representative. n = 3 of sets of 30 seedlings each. Control was treated the same as experimental except not irradiated. **Seed germination (B)** and **hypocotyl elongation (C)** responses to exposure to 300, 305, 311, 317, 325, 332, 368 nm or to no UV (Control) irradiated 3 d after planting. For germination, scoring occurred 3 d after irradiation for seed/seedling maintained in complete darkness (Dc, except for irradiation period) or 16∶8 long day (W_LD_). For hypocotyl experiments, scoring took place 5 d post irradiation. Standard deviation is shown, and significance is indicated on the Figure by P values on 1B, and stars on 1C, where **** indicates P values <.0001, *** indicates P value<.001, **P value<.01, and * P value in that instance shown is 0.05.

As a first step to assess UV responses, young soybean seedlings were irradiated with specific UV-A and UV-B wavelengths, then the primary leaves were extracted for absorbance spectra (Fig. 1A). A pulse of 300, 317 or 368 nm treatment was delivered overhead to 4 d dark-grown seedlings, then the primary leaf pair was harvested 24 h after irradiation for extraction in aqueous solution, and absorbance read. Each wavelength produced unique spectra, however 300 nm treatment produced a spectrum similar to the Dark Control (mock irradiated, no light).

Germination (breaking testa, emergence of radicle) was impacted by exposure to UV radiation in a wavelength-dependent manner, observed 3 d after irradiation, where sets of seeds were maintained either in complete darkness (Dc), or in Long Day white light (W_LD_) (Fig. 1B). Compared to unirradiated controls, seeds exposed to UV-B 300, 305, or 311 nm, then maintained in Dc, had greatly reduced germination (≤47%, compared to 99% for control) but were not significantly different from each other (P>.05). Exposure to 317 nm, in the intermediate range between UV-A and UV-B, germination was scored ∼60% in Dc, and was significantly different from 311 nm treatment (p = .0116). Germination in response to 325 and 332 nm was scored over 80% for both wavelengths, where germination rate was significant in comparing 325 to 317 nm values, and 325 and 332 nm germination values (shown on Fig. 1B). Comparisons of both the germination rates for 332 and 368 nm were not significant, and 368 nm compared to dark unirradiated controls (Dc control) similarly were not significant (P>.05).

The reduced germination effects of UV observed at all wavelengths at 317 nm and below was mitigated (germination rates increased) by placing seeds into a long-day 16∶8 LT::DK cycle (W_LD_) immediately following UV exposure (Fig. 1B). Interestingly, in W_LD_ conditions, some individual wavelength comparisons were not significant, as if the germination rate was step-wise in plateau. For example, germination rates of 300 compared to 305 nm, 317 to 325 nm, and 332 to 368 nm, were not significant; and, 332, 368 nm and W_LD_ controls were not significant in comparison (P>.05). Other pairwise comparisons were significantly different (shown on Fig. 1B) in germination if seedlings where grown under W_LD_, post-irradiation. A 2-way ANOVA comparing Dc and W_LD_ data across all wavelengths indicates significance (P<.0001).

Hypocotyl responses measured 5 d after irradiation did not mirror the germination Dc to W_LD_ differences. Elongation was progressively more impacted at shorter wavelengths (Fig. 1C) in the Dc condition, and was linear. If each wavelength was compared to the next greater (i.e. 300 to 305 nm, 305 to 311 nm etc) the significance for each pair was P>.0001, except for Dc 368 nm compared to the Dc control (no irradiation) where P = .0285. Hypocotyl elongation increased with each increasing wavelength in the W_LD_ condition, except that the hypocotyl elongation response plateaued by 332 nm. In specific pairwise comparisons of each wavelength, all hypocotyl lengths of Dc compared to W_LD_ in response to a specific UV wavelength were significantly longer (P values pairwise, 300 Dc to 300 W_LD_ etc.), where P values ranged from 0.0468 to <.0001 (significance indicated on Fig. 1C), except for elongation in response to 332 nm which did not reach significance in Dc compared to W_LD_. Due to the plateau of response in the W_LD_, the hypocotyl length of 368 Dc compared to 368 W_LD_ was significantly different (P<.0001), and hence, 332 and 368 nm in W_LD_ expanded to a similar length as the W_LD_ control seedlings and was not significantly different.

### Ultrastructural responses to UV radiation

**Figure 2:**
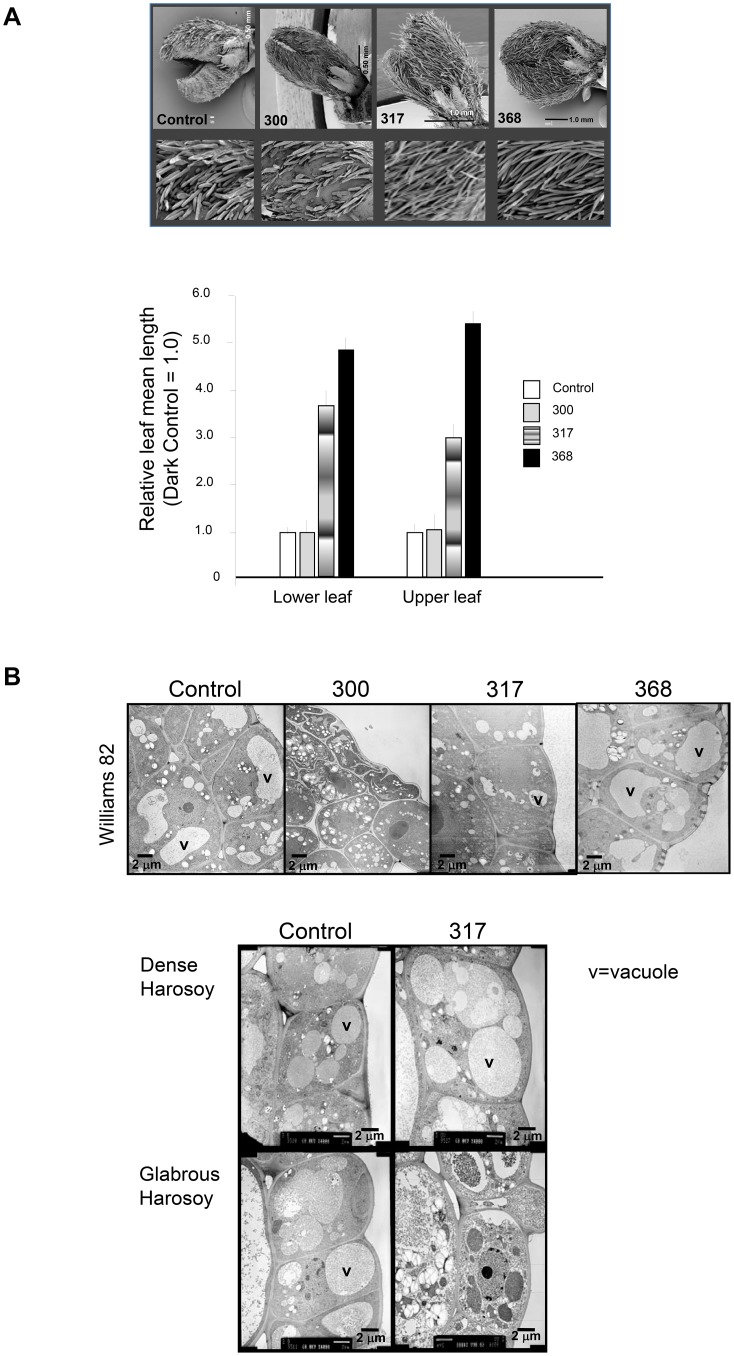
Representative SEM and TEM images of UV-A and UV-B responses. A. SEM indicate developmental response to UV-A and lack of, or inhibited response to 300 nm for development of pubescence. 4-d-old seedlings were mock-irradiated (Control) or irradiated with a brief pulse of UV (300, 317 or 368 nm), then returned to the dark for 24 h after which the primary leaves were harvested directly for Scanning Electron Microscopy. Views of the primary leaves of seedlings indicate the side views of the primary leaf pair. The lower panel depicts representative enlarged insets of the side views of the primary leaves showing the rows of hairs. Irradiation treatments are indicated in the Figure. The bar graph depicts the mean length of 10 randomly selected hairs from what will later (after opening) be the abaxial side, with the mean value shown. Mean value of Control seedlings’ primary leaves was set to 1.0, and other treatments thus compared to Control. **B. TEM micrographs of etiolated 5-d-old soybean seedlings grown exposed to UV indicate damage at UV-B wavelengths.** 4-d-old Williams 82 (the cultivar used throughout unless indicated) seedlings were either mock-irradiated (Control) or irradiated with 10^4^ µmolm^−2^ of UV (300, 317 or 368 nm), then returned to the dark for 24 h, after which the primary leaves were harvested directly into fixative in the dark to be processed for TEM. Sections were cut perpendicular to the abaxial (top, emergent side of leaf) surface and show the side view of the epidermis and first developing mesophyll cell layer. Representative sections are shown. v = vacuole. Second set (panels below). Soybean cv Harosoy dense (top row) and Harosoy glabrous (bottom row) were sown and grown the same as for Williams 82. The 4-d-old seedlings were either mock-irradiated (Control) or irradiated with 10^4^ µmolm^−2^ of UV (317 nm), then returned to the dark for 24 h after which the primary leaves were harvested directly into fixative in the dark to be processed for TEM. Sections were cut perpendicular to the abaxial surface, and representative sections are shown. v = vacuole.

With UV clearly having tissue-dependent impacts, some detrimental, some growth promoting, we further investigated the primary leaves since their development for photosynthesis is critical to overall seedling survival. Primary leaves of control, and UV-treated seedlings were examined by SEM. Control 4-d-old (mock-irradiated) seedlings, and seedlings exposed to UV-B radiation (300 nm) exhibited organized rows of densely packed, but unexpanded leaf hairs 24 h post-irradiation (Fig. 2A), with some hairs appearing damaged in response to 300 nm. In contrast, the pubescence on primary leaves of seedlings treated with 317 nm or 368 nm expanded, covering the surface of the primary leaf, with 368 nm eliciting the greatest response on the upper leaf (exposure side). TEM data of the primary leaf pair revealed cellular damage as a result of the 300 nm (UV-B radiation) treatment including organellar damage, membrane damage and the loss of cell wall integrity in both the epidermal layer, and developing mesophyll layer just below the epidermis (2B, upper set of images). Despite having an apparent developmental response to 317 nm (pubescence), this treatment resulted in some vacuolar disruption observed in the TEM, but there was no visible loss of plasma membrane/cell wall integrity. The 368 nm treatment (UV-A) appeared to promote plastid development (compared to proplastids in Controls) with no overt deleterious effects on the cells.

To confirm the SEM and to explore whether leaf hairs have a specific role in protecting young etiolated leave cells from UV radiation, we did TEM to explore the effects of 317 nm treatment on the isogenic Harosoy dense (pubescent/hairy; L62-801) and glabrous (bald; L62-561) cultivars of soybean at the same age (4-d-old), by examining the epidermal and immediate adjacent layer of developing mesophyll of the primary leaf pair (2B, lower set of images). The TEM data indicated that the dense (hairy) line displayed no obvious deleterious effects in response to treatment with 317 nm radiation. In contrast, the glabrous (hairless) line displayed severe damage, including vacuolar and organellar damage, plasmolysis, and loss of plasma membrane and cell wall integrity.

### UV stimulates expansion of pubescence and pigments at some wavelengths

**Figure 3:**
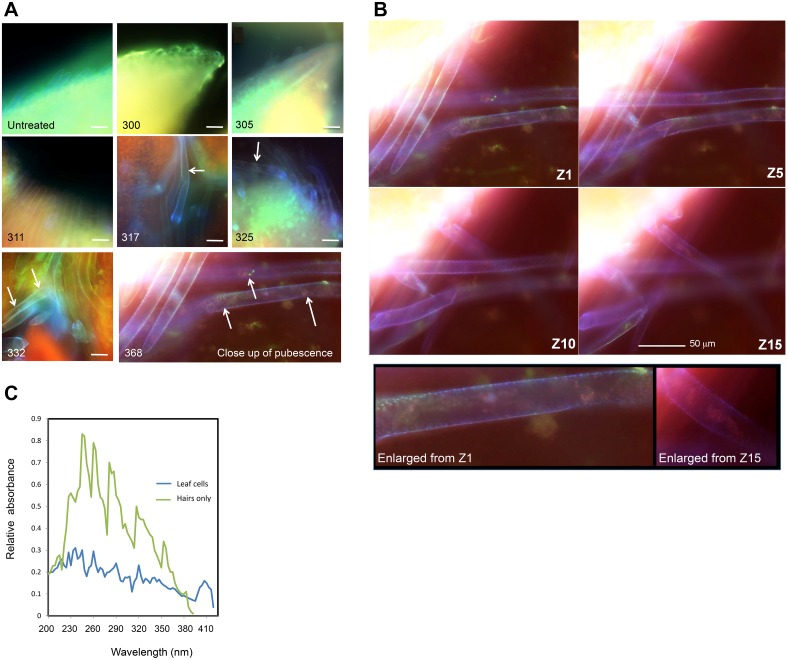
UV- and visible-light absorbing compounds are located in pubescence, induced by UV-A or UV-B or Phe. A. Responses of live-seedling pubescence to UV-B and UV-A wavelengths on deconvoluting microscopy. 4-d dark grown seedlings were either mock-irradiated (Untreated) or irradiated with 10^4^ µmolm^−2^ of UV (300, 305, 311, 317, 325, 332 or 368 nm shown), then returned to the dark for 24 h, after which the live primary leaves were imaged on a deconvoluting microscope. The images shown are captured (false-colored) for DAPI (blue), FITC (green) and TexasRed (red) excitation/emission sets (merged images shown) and show natural fluorescence, and represent a snap of the Z-stack focused on the abaxial pubescence of the primary leaf. At least 30 seedlings were viewed per condition. A close-up of the pubescence from 368 nm treatment is shown. Scale bar represents 40 µm. Arrows indicate the increasing (compared to Untreated) fluorescence. **B. Z-stack representative slices of fluorescence of 368 nm treatment.** Each slice of the Z stack = 1 µm thickness. **C. Spectra of leaf material compared to pubescence.** Soybean were grown in complete darkness, irradiated with the wavelength as shown on the Figure at 4-d-old, then primary leaf pairs were harvested from seedlings into liquid nitrogen, where pubescence was separated then extracted for aqueous UV-absorbing molecules. Absorbance was read from 200–420 nm. Spectra are representative. n = 3 of sets of 30 seedlings each.

Deconvoluting fluorescence microscopy was used to further evaluate the pubescence of live etiolated seedlings in response to a UV treatment 24 h post-irradiation. In order to view pigmentation in the live leaves and resulting pubescence, excitation and emission filter sets were used that span UV and most of the visible spectral region. A brief UV-B treatment (300, 305 or 311 nm) did not increase fluorescence intensity or elongation of pubescence of live seedlings (Fig. 3A). Response to 300 and 305 nm indicated that hairs remained short or even damaged (300, hairs appear folded). As observed in SEM data (Fig. 2A), seedlings treated with 317 nm also exhibited expanded pubescence, and additionally, pigments were observed within the pubescence. Fluorescence was more visible in seedlings treated with UV-A radiation (325, 332 or 368 nm) (Fig. 3A & B), and for detail Figs. 3B and S1 indicate slices through the pubescence of the seedlings with the most visible pubescence, those seedlings treated with 368 nm. In particular, the DAPI and FITC (exciting in UV and blue, and blue and green, respectively, with emission in blue and in green, respectively) channels indicated the predominant emissions, which would fit with what is known about phenylpropanoid absorbance. Absorbance spectra of primary leaves confirm that 300 nm elicits little in the way of synthesis or deployment of UV-absorbing compounds compared to 317 or 368 nm (Fig. 1A). Since pigments were noticed at 317 nm, and 317 also had a similar absorbance spectrum to 368 nm, analysis was performed on the hairs treated with 317 and compared to the rest of the leaf material. Pubescence was separated from the primary leaves 24 h after being treated with 317 nm, then the pubescence was further analyzed by absorbance spectra (Fig. 3C) and HPLC for specific phenylpropanoids. There were many compounds that were too low in abundance to be detected in the hairs by these methods, but of individual compounds detected, sinapic acid esters were observed to have the greatest increase compared to pubescence of untreated primary leaves (0.83 ng/g [317 nm] compared to 0.03 ng/g [untreated], S2 Fig.).

### Exogenous Phe prevents any negative effects of UV-B

**Figure 4:**
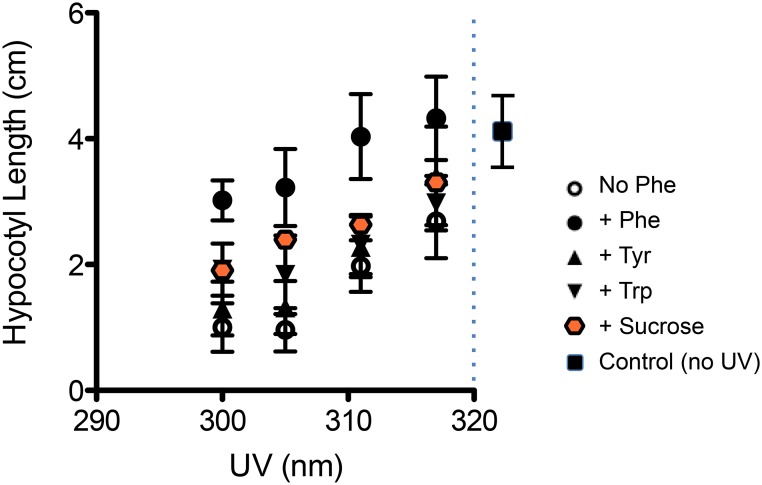
Phe prevents deleterious effects of UV-B, not duplicated by tryptophan, tyrosine or sucrose. Images of seedlings grown and exposed to UV radiation (300, 305, 311, 317) as described in Fig. 1, except that seedlings were grown on media with (+) and without (–) inclusion of 500 µM Phe, or 500 µM tryptophan (Trp) or 500 µM tyrosine (Tyr), or 2% final volume sterile-filtered sucrose (Sucrose) at the time of seed sowing. Hypocotyls were measured in cm 5 d after irradiation. Symbols are indicated on the Figure. Some of the symbols plotted are obscured, as there were nearly coinciding data points (i.e. tryptophan symbol is right behind sucrose for the 300 nm treatment).

Phenylpropanoids can absorb light in 300–415 nm range, and are the basis of the most commonly observed responses (screening) to UV of field plants in the literature. Phe also is the precursor to many structural molecules involved in defense responses of seedlings. Hence, we explored how exogenous Phe would affect particular responses to UV-B which we had shown in Fig. 1 to be harmful at more energetic wavelengths (<317 nm). Addition of Phe (1.0 mM) to the growth media at the time of sowing resulted in 100% germination and greening by 5 d post-irradiation, compared to UV-B treated seedlings with no exogenous Phe added (S3A Fig.). Hypocotyl elongation data (S3B Fig.) were similar in the unirradiated control, and irradiated seedlings for all UV-B treatments when Phe (1.0 mM) was added to the media. Since 1.0 mM appeared to saturate this ‘prevention of damage’ response, we also utilized other concentrations of Phe in a similar set of experiments, shown in Figs. 4 and S4. The more energetic wavelengths (300, 305 nm) still caused suppression of elongation, but not as severe as –Phe. To explore if this prevention of damage was specific to Phe, we tested similar amino acids, or amino acids thought to regulate Phe in other organisms, in order to see if another amino acid could impart the same protective effects (Fig. 4). Neither tyrosine nor tryptophan at 500 µM could duplicate the effect of Phe, however there was some response observed, where tryptophan had greater hypocotyl elongation at 300 nm compared to 305 or 311. Tryptophan itself, unlike Phe, is known to be able to directly absorb UV directly ∼300 nm; conversely, tyrosine has low absorbance at 300 nm. To further clarify the mechanism of Phe’s action, 2% sucrose was included in the top agarose at time of sowing, in case the hypocotyl alleviation response was due to a specific carbon issue, as the germinating seedlings do not have a developed or mature chloroplast. Sucrose also failed to alleviate the hypocotyl suppression of UV-B in the more energetic wavelengths. While 100 µM of Phe impacted the protective effect and promoted elongation, it was not as great as 500 µM Phe. 100 µM tyrosine or tryptophan did not improve or alter responses to UV (S4 Fig.).

### Phe and UV-B can increase development in the primary leaves

**Figure 5:**
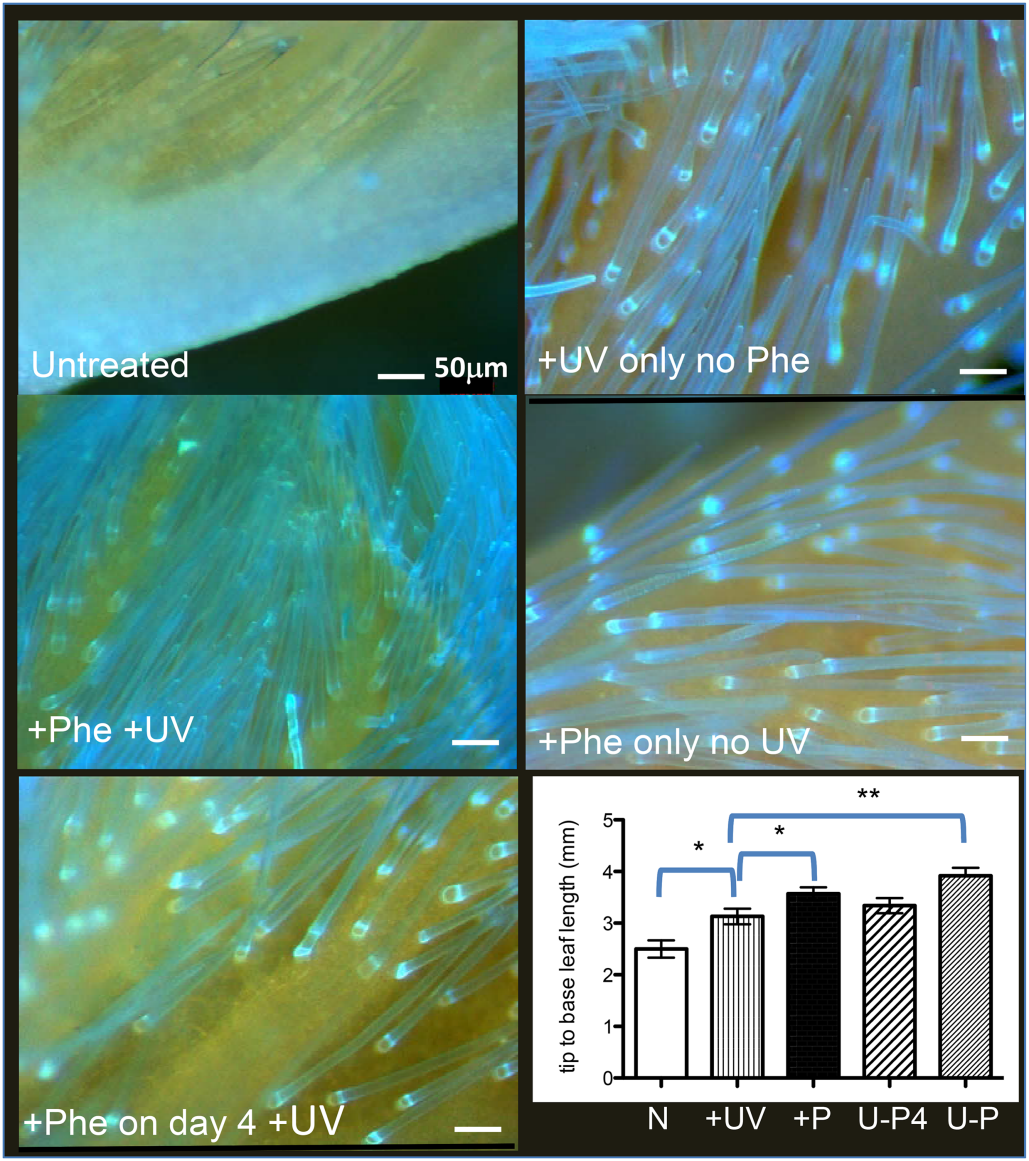
Primary leaves respond to Phe or UV (317 nm): impact on pubescence and leaf expansion. All data shown are from 5-d-old seedlings (4-d-old seedlings were irradiated with 317 nm, returned to darkness, then images captured 24 h later), and images were taken on a Stereo dissecting microscope utilizing a DAPI-longPass cube set, with excitation peak at 325 nm and emissions that covered the full visible spectrum (in real color). Images were captured at the same exposure. The Control (“Untreated” image; “N” tip to base leaf length) seedlings were not treated by any UV irradiation, and the medium did not contain Phe at any time. “+Phe +UV” image, “U-P” tip to base leaf length indicates data set where Phe was included in the medium from sowing, and 4-d-old seedlings were irradiated with 317 nm then returned to darkness and harvested 24 h later. “+Phe only no UV” image; “+P” tip to base leaf length were seedlings treated with Phe alone in the medium from sowing, harvested at the same time as all other seedlings (24 h irradiation times). “+UV only no Phe” image, “+UV” tip to base leaf length are seedlings receiving a 317 nm UV irradiation without any Phe at any time, returned to darkness and harvested 24 h post-irradiation. “+Phe on day 4 +UV” image; “U-P4” tip to base leaf length indicate seedlings grown in the same manner as controls, until 4-d-old seedlings, where Phe was added to the medium 1.0 h before the UV (317 nm) irradiation, returned to darkness, then viewed 24 h later. All seedlings thus were the exact same age (5-d-old). The red flourescence indicates protochlorophylls and developing plastids from developing mesophyll under the epidermis. n = 30 seedlings each condition. The scale bar represents 50 µm. (* = P<.05; ** = P<.01).

Data herein indicate Phe and UV have developmental impacts on the germinating and young soybean seedling. Since Phe-derived materials can be up to a third of the vegetative mass of a plant [44], and Phe synthesis is known to be increased by UV by many reports, it is possible that there is some interactive effect of Phe and UV in the context of leaf and/or pubescence development. In order to investigate this further, we examined pubescence and leaf expansion on a dissection microscope utilizing DAPI LongPass capture. Viewed samples are excited at a peak of 325 nm (excitation occurs in both UV-A and UV-B) and the emission from the plant material is captured at UV-A and all visible wavelengths. When Phe was included in the medium from sowing, and 4-d-old seedlings were irradiated with 317 nm then returned to darkness and harvested 24 h later, an increase on pubescence density, fuorescence and leaf expansion was observed (Fig. 5; “+Phe +UV” image, “U-P” tip to base leaf length), compared to seedlings treated with Phe alone in the medium from sowing (“+Phe only no UV” image; “+P” tip to base leaf length), or a UV irradiation without Phe (“+UV only no Phe” image, “+UV” tip to base leaf length). The U-P leaf expansion measurement indicated the +Phe and +UV impact was not directly additive. If Phe was added to medium of 4-d-old seedlings, 1.0 h before the UV (317 nm) irradiation, then viewed 24 h later (“+Phe on day 4 +UV” image; “U-P4” tip to base leaf length), there was little change in response observed, compared to UV or Phe alone, so Phe itself directly is not absorbing UV to fluoresce in the pubescence. The Control (“Untreated” image; “N” tip to base leaf length) seedlings which were not treated by any UV irradiation, and the medium did not contain Phe at any time remained non-fluorescent (clear) and leaves relatively unexpanded. The pubescence itself is largely non-flourescing (without Phe or UV) compared to the cells of the cell layer below, which are fluorescing in the red region, indicating presence of protochlorophylls/chlorophyll.

## Discussion

The results of this study illustrate wavelength-dependent effects of exposure to UV radiation, and how Phe can directly influence the seedling response outcome. Each parameter assessed had a unique action spectrum for UV, where for some, there was a distinct difference between UV-A and UV-B responses (germination and maintenance in darkness; pubescence fluorescence, absorbance of aqueous extract of primary leaves), and some parameters appeared to respond in a near linear fashion to increasing wavelength (germination moved to W_LD_; hypocotyl elongation moved to Dc) underlying potentially shared as well as unique mechanisms of signal transduction. We demonstrated that Phe can prevent a stressful response or cessation of growth response, preventing deleterious responses in development/greening, germination, and elongation. Compared to tyrosine and tryptophan, Phe itself does not absorb well in the UV-B range, However, the products of the phenylpropanoid pathway are utilized for UV-screening, as well as structures of protection made from phenylpropanoids, and further, Phe may have yet other uses not yet known for the seedling. Phe facilitates a robust response to UV via the phenylpropanoid family, and these actions are not duplicated by tyrosine or tryptophan. Phe in a variety of contexts is important for the germinating, then development in the seedling, illustrated in the combined response of Phe and UV effects on pubescence, while not additive was an increase over +Phe alone or UV exposure alone, as shown herein.

Although the goal of this study was not to develop an action spectrum *per se*, the featured results are consistent with the steep UV-B response observed in several commonly cited biological spectral weighting functions such as those of Caldwell (1971) [45] and Flint *et al.*, 2004 [46], and the DNA damage spectrum of Tan *et al.*, (1970) [47]. Likewise, the UV-A induced responses were also consistent with those previous studies where negative effects of UV-A were reduced compared to UV-B (i.e. more flat response) and with previous studies that indicated the physiological importance of UV-A and its role in gene expression and phenolic accumulation [48], [49].

Given that our studies focused on seeds and very young seedlings, some in complete darkness from the time of planting, Phe utilization early in the germination-to-seedling transition may be a general mechanism of UV-preparedness in the absence of a fully functional chloroplast. Indeed, studies of UVR8, a known UV-B receptor, indicate that mutation of a single tryptophan to Phe enables a re-tuning of the photoreceptor to be able to detect UV-C wavelengths [7]. Phe is an important building block, is the starting point of the phenylpropanoids, and may assist in screening more energetic (below 280 nm) wavelengths of UV, which was not tested herein, but others have observed that there are UV-absorbing pigments induced in the low UV-B and UV-C ranges [50]. The utilization of Phe demonstrated herein indicates that Phe may be specifically required to supply the rapidly growing ‘sink’ of the new leaves, chloroplasts, and defense structures.

### UV effects on germination and growth

Seed germination was reduced by exposure to UV-B, but exposing seeds to low levels of PAR (W_LD_) following UV exposure reversed the UV-B inhibition of seed germination. Since germination in dark-grown control seeds was ∼100%, it is not likely that the response to light was simply a function of light-induced germination. The mitigation of UV-B damage by high levels of PAR has been known for some time (e.g. [28], [29] and others), but the responses observed herein suggest that the response is elicited at very low levels of PAR (e.g. 10^2^ µmol m^−2^ s^−1^ in this study). The mechanisms remain poorly understood. In this study with low levels of PAR and etiolated seedlings it is not likely that photosynthetic carbon gain or available energy was a factor in the response. Since the germination rate varied with wavelength of UV even after subsequent exposure to white light, the response could be linked to a either a UV-white light-reversible photoreceptor or, more likely, a white-light-induced repair or reversal of some deleterious effects of the UV exposure.

Hypocotyl elongation was also affected as a function of wavelength in a similar fashion as germination. However, in contrast to germination, responses were linear (in germination compare Dc to W_LD_) and subsequent exposure to white light had very little impact on the response, except in the controls and at 368 nm where hypocotyl elongation was reduced in the light. These hypocotyl elongation data show a similar trend in the spectral responses to that reported by Gardner *et al.*, 2009 [4]. These data suggest that hypocotyl length may be a function of a different receptor(s) or different mechanism(s) of control than germination.

### Pubescence and UV-B Screening may be linked to Phe levels

The SEM, TEM, and deconvoluting microscopy data suggest that young etiolated soybean leaves perceive UV-A radiation as a developmental signal. This response may enhance UV protection of the nascent leaves by UV-absorbing compound synthesis, increasing leaf reflectance, or screening potentially damaging UV-B radiation by absorbance in the leaf hairs. Although leaf reflectance of UV-B is generally below 10%, it has also been shown to be up to 70% in some plants [51], [52]. Day [19] showed that herbaceous dicotyledons may permit penetration of UV-B >30 µm into the leaf surface, which would span the epidermis, and extend into forming mesophyll of soybean primary leaves in early seedling stage. Leaf hairs have been shown herein to accumulate UV-screening compounds, hence it is possible that the more dense isoline of Harosoy may possess more phenylpropanoids in strategic locations for defense compared to the glabrous line. Pubescence and leaf surface waxes affects leaf optical properties ([53]–[55] and can enhance tolerance to UV radiation [56].

### Importance of Phe in structures and screening molecules

The synthesis of UV-screening compounds in response to exposure to UV radiation and its role as an adaptive response of plants to UV radiation has been well documented [9], [13], [49], [57]–[59]. Most previous research has focused on the genes coding for synthesis of enzymes that define key steps in the phenylpropanoid pathway or its major branch points or terminal steps (e.g. PAL, CHS). Almost no studies have focused on the availability or synthesis of Phe, especially in the seed to seedling transition in seedlings, when seedlings are dependent on the contents of the seed and environmental signals.

Phe, synthesized by plants *de* *novo*, serves as both a building block for proteins, as well as the precursor or first step to the thousands of compounds synthesized in higher plants by the phenylpropanoid pathway. Levels of Phe can be low enough to limit the production of UV-protective compounds such as UV waxes and UV-screening pigments [3], [15]–[17], [41]. Depending upon the quality and quantity of the materials stored in the endosperm, emergent seedlings may not have sufficient Phe to serve primary metabolism (protein) and the ability to achieve UV-B protection, since only particular areas of the growing seedling (i.e. root tip) appear to possess measurable phenylpropanoids [60], [61] and the plastid is still developing. Voll et al. [42] reported the interesting finding that increasing carbon availability affected growth of plants that were being fed exogenous amino acids including Phe, but the relationship to plastid status is unclear.

The results of this study demonstrate that supplementing Phe in soybean prevents potential damage by UV, providing a competency to etiolated seedlings to respond to UV-B. The ability to prevent UV-B stress in etiolated soybean by the feeding of Phe to the imbibing seed in the absence of any pre-irradiation, suggests that a significant amount of Phe is committed to the phenylpropanoid pathway [3], [17], [62]. In addition, the significant and measurable chemical change in hairs in response to 317 nm was the increased accumulation of sinapate esters, a phenylpropanoid demonstrated to be important in UV screening (11,12). Hence, Phe may be a limiting factor in seedling responses to UV.

It is not known however whether soybean is similar to *Arabidopsis* mechanistically where exposure of etiolated *Arabidopsis* seedlings to BL or to UV-A radiation activates a G-protein-mediated pathway that increases the rate of synthesis of the amino acid Phe and subsequently increases UV radiation-absorbing pigments [3], [41]. The larger seed and seedling size in soybean compared to *Arabidopsis* may also affect the dynamics of seed/leaf optical properties and thus UV-screening may differ inherently between the two species. However, like Arabidopsis, it is possible that sinapate esters [11]–[12] are also very important in screening for soybean given our data herein for the pubescence. Day [19] and Day et al., [63] have shown that epidermal screening effectively varies widely among species and it is related to growth habit and ecological role. It is also unclear how these responses may vary in more mature plants. The 5-d-old seedlings in dark-grown conditions observed herein possess proplastids like other legumes [64], which are different from a mature seedling, or adult plant with developed chloroplasts. Light-grown, mature plants possess fully functioning chloroplasts, and presumably ability to synthesize Phe by various means.

### UV-exposure and acclimation to environmental UV

UV-A above 317 nm promoted strong developmental responses (pubescence, sinapate accumulation) with some indicators of stress or damage at 317 nm (vacuolar changes, Harosoy data). There were no apparent developmental responses and minimal pigment production at 300 nm, indicating there may be a range of photoreceptors spanning the UV-B range, and the photoreceptors concerned may be less efficient at higher energy wavelengths like 300 nm.

It is of interest to consider the presumed enhancement of UV-B tolerance or acclimation to UV-B via putative UV-A signal perception. Perhaps this can be rationalized by the fact that incident levels of UV-A are several orders of magnitude larger than those of UV-B in the natural environment, and the high degree of epidermal attenuation of UV-B in many species [19], [64], [65]. It could also be due to DNA damage at the shorter wavelengths. For example Buchholz *et al.* (1995) [66] suggested that UV-B inhibition of flavonoid synthesis may have been due in part to DNA damage since BL/UV-A induced photoreactivation partially reversed the inhibition. In plants exposed to UV-A and UV-B, there are likely interactions between UV-A and UV-B systems that we still have very little information regarding, as yet [67]. It would not be surprising if selective pressure led to UV-A rather than sole UV-B photoreceptors in plants not adapted to a very high UV environment (i.e. adapted to low altitudes). This would be somewhat analogous to the BL/UV-A induction of photolyase and the repair of cyclobutane pyrimidine dimers (CPD) [32], [68]. UV-A also stimulated greater accumulation of pigments in the pubescence that absorbed in UV (shown in Fig. 3).

The possible selective value of such a UV-A response is further evident when one considers the range of ecophysiological benefits such as improved drought and pest tolerance, and photoprotection is generally attributed to development of leaf pubescence and glaucousness [53], [69]–[73]. Phenylpropanoids in general are part of defense mechanisms, where adequate induction of the phenylpropanoid pathway is important in the first three weeks post-germination, influencing ability to respond to subsequent stresses [1], [74]–[77]. Hence, the level of Phe present in a germinating seed may be critical for the ability to acclimate to new environmental conditions. Further studies are needed in order to further elucidate the importance of initial levels of Phe and the interactions with development and environmental signaling modifiers.

## Supporting Information

S1 Fig **Z stack of 368** **nm-irradiated seedlings showing individual channels for the Z1 slice**. Seeds were planted as described for Fig. 3A. The image shown indicates the black and white contrast of the individual channels for DAPI, FITC, and Texas Red, indicating the impact of each excitation and emission. DAPI excites in UV and emission is in the blue range and is false-colored in blue, FITC excites in blue and blue-green, and emission is in blue-to-green range and is false-colored in green, Texas Red excites in the yellow to red, and emission is in orange to red.
(PPT)

S2 Fig **Mass Spectrometry (MS) chromatogram data for UV and untreated samples.** Arrow indicating peak of interest, sinapate, was confirmed by purified standard. Peak area was calculated by computer software described in Methods.
(DOCX)

S3 Fig **Phe prevents deleterious effects of UV-B on germination and hypocotyl elongation.** Seeds were planted as described for Fig. 1 except that in +Phe (1.0 mM) trays, Phe was included in the top agarose medium. On d 3 after sowing, the trays were irradiated with 300, 305, 311 or 317 nm as described in methods, then placed in WLD and photographed (**A**), scored and hypocotyls measured (**B**) 5 d later.
(PPT)

S4 Fig **Phe and aromatic amino acid impact on UV-irradiated hypocotyl length.** Images of seedlings grown and exposed to UV radiation (300, 305, 311, 317) as described in Fig. 1, except that seedlings were grown on media with (+) and without (–) inclusion of 1.0–500 µM Phe (upper panel), or 100 or 500 µM tryptophan (Trp) or 500 µM tyrosine (Tyr) (lower panel), Hypocotyls were measured in cm 5 d after irradiation. Symbols are indicated on the Figure. Some of the symbols plotted are obscured, by coinciding data points. Stars indicate significant differences (T-test, Welch correction) between the 100 and 500 µM Phe treatments (* = P<.05; *** = P<.001; **** = P<.0001).
(PPT)
