# Supplementary figures and images for: Phenylalanine Is Required to Promote Specific Developmental Responses and Prevents Cellular Damage in Response to Ultraviolet Light in Soybean (Glycine max) during the Seed-to-Seedling Transition
Source: PLoS One. 2014 Dec 30;9(12):e112301. doi: 10.1371/journal.pone.0112301 (PMC4280123; doi:10.1371/journal.pone.0112301)

## Slide 1
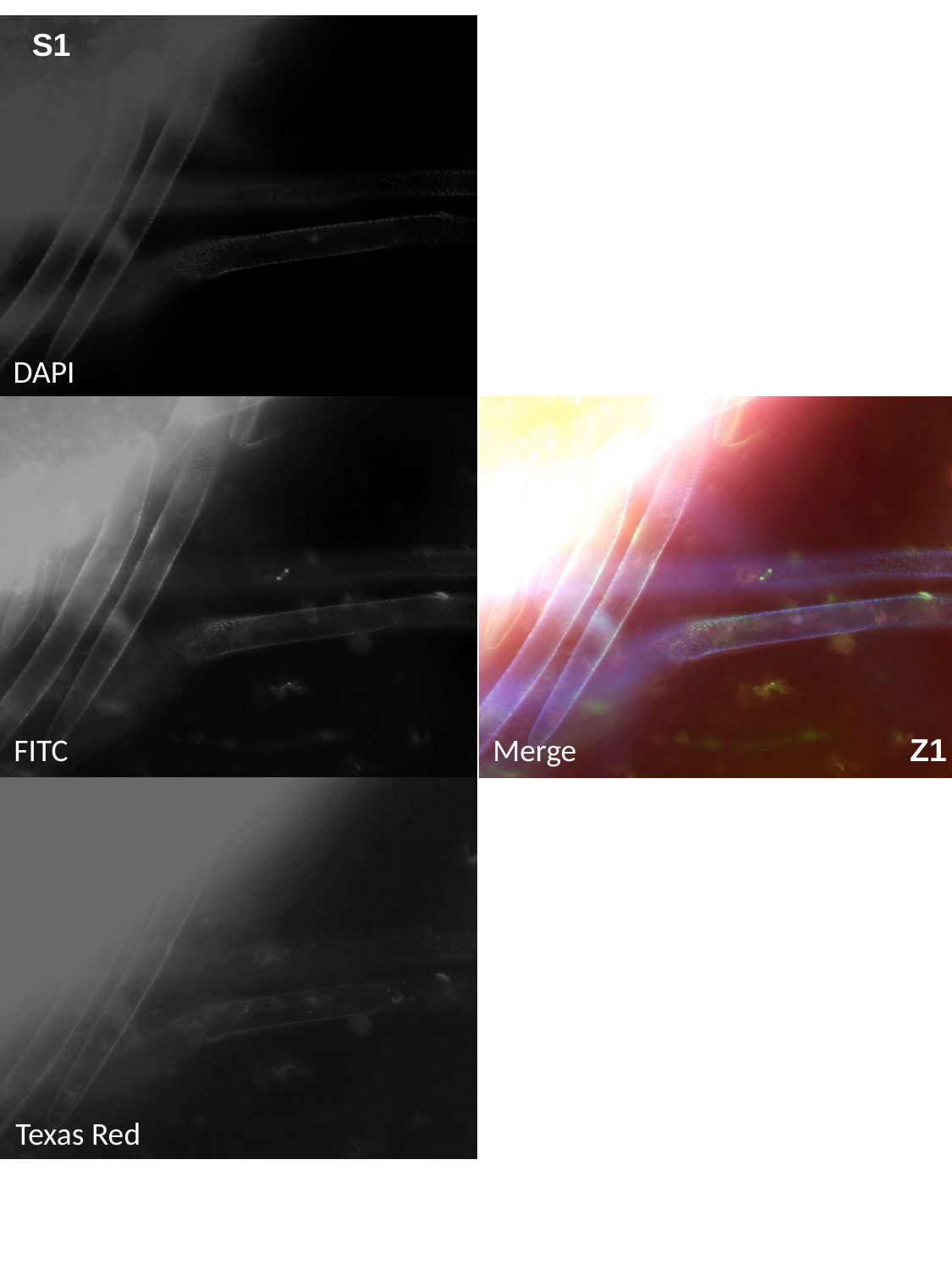

DAPI
FITC
Merge
Z1
Texas Red
S1

Supplement: S1 Fig — Z stack of 368 nm-irradiated seedlings showing individual channels for the Z1 slice. Seeds were planted as described for Fig. 3A. The image shown indicates the black and white contrast of the individual channels for DAPI, FITC, and Texas Red, indicating the impact of each excitation and emission. DAPI excites in UV and emission is in the blue range and is false-colored in blue, FITC excites in blue and blue-green, and emission is in blue-to-green range and is false-colored in green, Texas Red excites in the yellow to red, and emission is in orange to red. (PPT) [file pone.0112301.s001.ppt]

## Slide 1
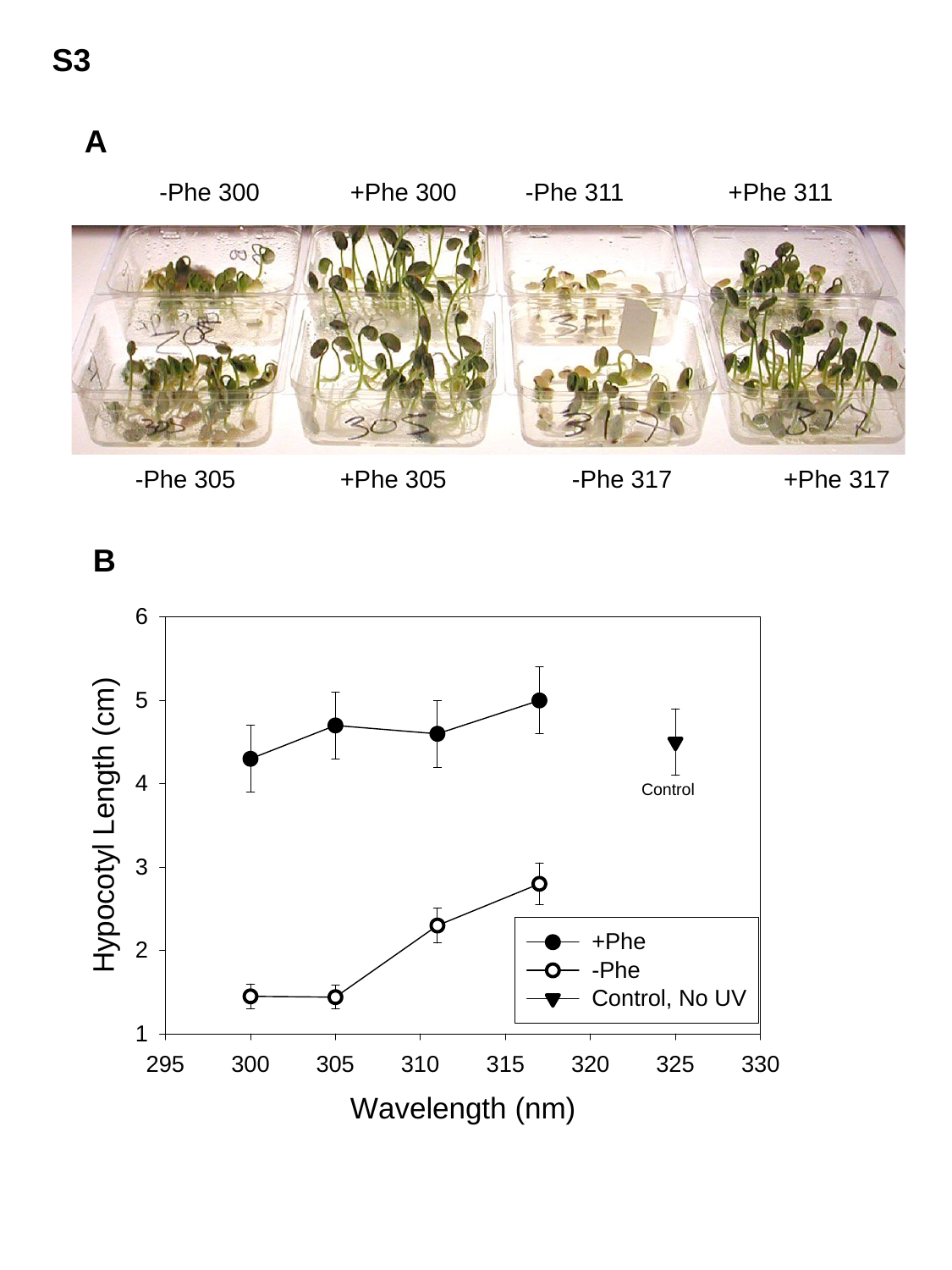

S3
A
-Phe 300 +Phe 300	 -Phe 311 +Phe 311
-Phe 305 +Phe 305 -Phe 317 +Phe 317
B

Supplement: S3 Fig — Phe prevents deleterious effects of UV-B on germination and hypocotyl elongation. Seeds were planted as described for Fig. 1 except that in +Phe (1.0 mM) trays, Phe was included in the top agarose medium. On d 3 after sowing, the trays were irradiated with 300, 305, 311 or 317 nm as described in methods, then placed in WLD and photographed (A), scored and hypocotyls measured (B) 5 d later. (PPT) [file pone.0112301.s003.ppt]

## Slide 1
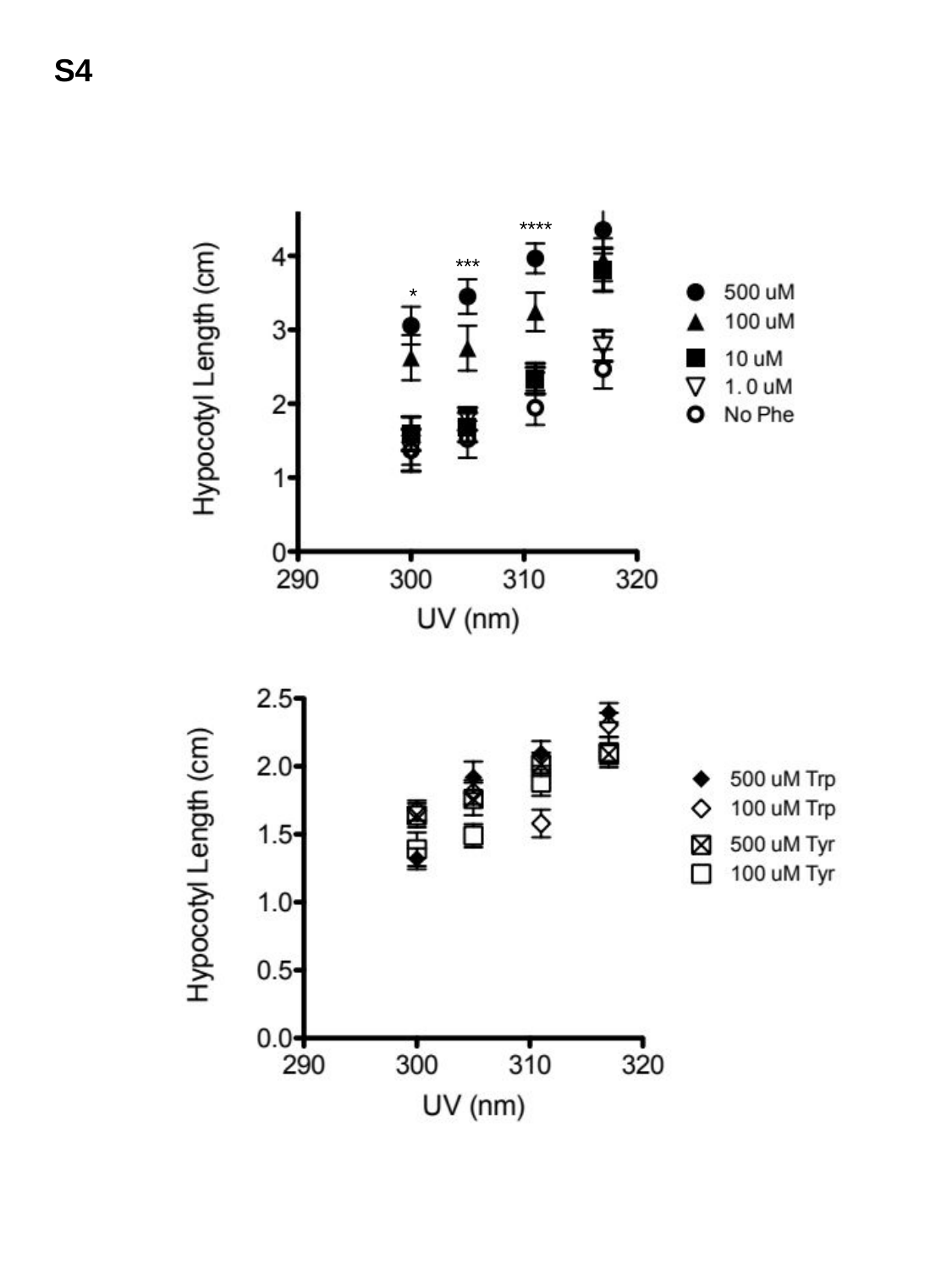

S4
****
***
*

Supplement: S4 Fig — Phe and aromatic amino acid impact on UV-irradiated hypocotyl length. Images of seedlings grown and exposed to UV radiation (300, 305, 311, 317) as described in Fig. 1, except that seedlings were grown on media with (+) and without (–) inclusion of 1.0–500 µM Phe (upper panel), or 100 or 500 µM tryptophan (Trp) or 500 µM tyrosine (Tyr) (lower panel), Hypocotyls were measured in cm 5 d after irradiation. Symbols are indicated on the Figure. Some of the symbols plotted are obscured, by coinciding data points. Stars indicate significant differences (T-test, Welch correction) between the 100 and 500 µM Phe treatments (* = P<.05; *** = P<.001; **** = P<.0001). (PPT) [file pone.0112301.s004.ppt]
