# Supplementary material for: Phenylalanine Is Required to Promote Specific Developmental Responses and Prevents Cellular Damage in Response to Ultraviolet Light in Soybean (Glycine max) during the Seed-to-Seedling Transition
Source: PLoS One. 2014 Dec 30;9(12):e112301. doi: 10.1371/journal.pone.0112301 (PMC4280123; doi:10.1371/journal.pone.0112301)
Supplement: S2 Fig — Mass Spectrometry (MS) chromatogram data for UV and untreated samples. Arrow indicating peak of interest, sinapate, was confirmed by purified standard. Peak area was calculated by computer software described in Methods. (DOCX) [file pone.0112301.s002.docx]

Supplemental Figure S2.

**UV sample**

Sinapic acid/Sinapate

**Untreated sample**

Sinapic acid/Sinapate

MRM in negative mode was used to detect compounds that appeared of interest.

Sinapic acid, in low abundance was detectable and increased in UV irradiation. The molecular weight is 224. In negative mode you subtract one unit, where sinapic acid runs at 223.

Standard:

1. MS-run a mass spectrum to see sinapic acid

1. MS2 – 223 peak was run in the mass spectrometer to see the breaking pattern.

1. An intense peak from above breaking pattern was chosen, 149, for the MRM breaking pattern. The MS selected ions of original mass 223, which breaks into 149 and detected the pattern, unique for this compound.
2. Peak area of the each MS chromatogram was used for quantitation. Standard calibration curve was used for the quantification of the samples.
